# Blood eosinophils to guide inhaled maintenance therapy in a primary care COPD population

**DOI:** 10.1183/23120541.00606-2021

**Published:** 2021-02-07

**Authors:** Helen F. Ashdown, Margaret Smith, Emily McFadden, Ian D. Pavord, Chris C. Butler, Mona Bafadhel

**Affiliations:** 1 Nuffield Dept of Primary Care Sciences, University of Oxford, Oxford, UK; 2 NIHR Oxford Biomedical Research Centre, Oxford University Hospitals NHS Foundation Trust, Oxford, UK; 3 Nuffield Dept of Medicine, University of Oxford, Oxford, UK

## Abstract

Blood eosinophils are a potentially useful biomarker for guiding inhaled corticosteroid (ICS) treatment decisions in COPD. We investigated whether existing blood eosinophil counts predict benefit from initiation of ICS compared to bronchodilator therapy.

We used routinely collected data from UK primary care in the Clinical Practice Research Datalink. Participants were aged ≥40 years with COPD, were ICS-naïve and starting a new inhaled maintenance medication (intervention group: ICS; comparator group: long-acting bronchodilator, non-ICS). Primary outcome was time to first exacerbation, compared between ICS and non-ICS groups, stratified by blood eosinophils (“high” ≥150 cells·µL^−1^ and “low” <150 cells·µL^−1^).

Out of 9475 eligible patients, 53.9% initiated ICS and 46.1% non-ICS treatment with no difference in eosinophils between treatment groups (p=0.71). Exacerbation risk was higher in patients prescribed ICS than those prescribed non-ICS treatment, but with a lower risk in those with high eosinophils (hazard ratio (HR) 1.04, 95% CI 0.98–1.10) than low eosinophils (HR 1.19, 95% CI 1.09–1.31) (p-value for interaction 0.01). Risk of pneumonia hospitalisation with ICS was greatest in those with low eosinophils (HR 1.26, 95% CI 1.05–1.50; p-value for interaction 0.04). Results were similar whether the most recent blood eosinophil count or the mean of blood eosinophil counts was used.

In a primary care population, the most recent blood eosinophil count could be used to guide initiation of ICS in COPD patients. We suggest that ICS should be considered in those with higher eosinophils and avoided in those with lower eosinophils (<150 cells·µL^−1^).

## Abstract 2

**ICS-naïve COPD patients who start on ICS and whose most recent blood eosinophil count is <150 cells·µL^−1^ have a 15% higher risk of future exacerbation compared to those with a count ≥150 cells·µL^−1^. ICS should be reserved for those with higher blood eosinophils.** https://bit.ly/3CiEFb9

## Introduction

Guidelines for pharmacological management of COPD recommend the addition of inhaled corticosteroids (ICS) to bronchodilator therapy for worsening symptoms (frequent exacerbations or persistent breathlessness) [1]. Although there is some benefit in reducing exacerbations, long-term effects of ICS on lung function decline and mortality are unclear. ICS use is associated with adverse effects including pneumonia and osteoporotic fractures, as well as being cumulatively expensive [2, 3]. Nevertheless, ICS compounds are widely used in clinical practice [4]. In UK primary care, almost two in five patients prescribed an ICS did not meet criteria for this treatment [3]. There is therefore an urgent need to improve clarity around when ICS should be prescribed.

Blood eosinophil count has gained interest as a biomarker for identifying COPD patients more likely to benefit from ICS treatment. Many *post hoc* analyses, using various eosinophil count thresholds, have shown greater response to ICS-containing preparations in patients with a higher baseline blood eosinophil count [5]. In addition, there have been recent prospective evaluations of ICS response and peripheral blood eosinophil count [6, 7]. However, patients included in such trials are not representative of the real-world population, as inclusion is centred around patients already established on inhaled maintenance medication. This is especially important in primary care, where patients are often diagnosed and where step-up or initiation of ICS therapy is most often considered [8, 9].

General practice research databases, which routinely collect anonymised information from patient consultations and are linked at patient level with hospital and national statistics, provide an efficient and well-validated way of answering clinical questions relevant to primary care involving large sample sizes [10].

Using the Clinical Practice Research Datalink (CPRD), we investigated whether the most recent peripheral blood eosinophil count at the point of an inhaled treatment step-up or initiation decision could predict treatment outcomes, in a COPD ICS-naïve primary care population from 2005–2015.

## Methods

### Study design

We used a new-user active-comparator study design [11] and compared time to first exacerbation of those commencing inhaled maintenance medication containing an ICS (ICS group) with those not containing an ICS (non-ICS group), looking for effect modification by baseline blood eosinophil count. Additional methods are presented in the supplementary material.

### Data source and included population

The CPRD, a large database of UK general practice clinical records, individually linked with Hospital Episode Statistics records, was used. Included patients were those with data linkage aged ≥40 years with a COPD diagnosis code, a valid blood eosinophil count (see later for definition), a history of current/past smoking and spirometry diagnostic of COPD (forced expiratory volume in 1 s/forced vital capacity ratio <0.7), who were starting a new inhaled maintenance medication for COPD in the period 1 January 2005 to 31 August 2015 (index date). Our range of index dates were chosen to be after introduction of Quality and Outcomes Framework targets in UK primary care which improved coding of COPD and spirometry [12], but before blood eosinophils were promoted as a potential biomarker, which might have influenced prescribing choices. In addition, recruited patients were ICS-naïve, due to concern that steroid treatment might suppress blood eosinophil values [13], which was defined as no ICS prescriptions and fewer than three oral corticosteroid prescriptions in the previous 12 months. Excluded patients were those with a diagnosis of bronchiectasis, alpha-1 antitrypsin deficiency, interstitial lung disease or cystic fibrosis. Those with an active diagnosis of asthma (coded in the past 2 years *versus* a historical code on the medical records) were excluded from the primary analysis. Follow-up continued until the earliest of the date the practice stopped providing data to CPRD, the patient died or left the practice or 29 February 2016. Eligible patients were required to have ⩾24 months continuous data, 6 months before and after the index date, to ensure adequate recording of baseline covariates and outcomes.

### Exposures

The exposure was a new ICS-containing inhaled maintenance medication (ICS, ICS/long-acting β_2_-agonist (LABA), or ICS/long-acting muscarinic antagonist (LAMA)), compared with a non-ICS treatment (LABA, LAMA or LAMA/LABA). A new inhaled maintenance medication was defined as a prescription for that drug category that had not been issued in the previous 12 months. Those commencing triple therapy were excluded to enable a better comparison between ICS and non-ICS therapies, and to minimise confounding, as in similar studies [14]. While outside guidelines, prescriptions for ICS monotherapy were included, as this is a common initial treatment for COPD in other database studies [15] and we wanted to reflect real-life practice. Designated prescriptions had to be continued for a minimum of 6 months after the index date (for the primary analysis). Continuous use was defined as treatment duration totalling ⩾90 days’ supply, similar to methods used in a previous study of ICS in COPD [14]. Patients that had a change or addition of another inhaled medication within 30 days of the index date which would result in a change of comparator group were excluded. Sensitivity analyses (supplementary material) explored the effect on results of managing medication adherence and changes of drug class.

To examine a potential dose–response relationship in ICS-containing medications, the strength of ICS prescribed on the index date was stratified into low, medium and high (corresponding to estimated equivalent daily doses of beclomethasone dipropionate of ≤500 µg, >500–1000 µg and >1000 µg, respectively), as used elsewhere [14], but higher than standard clinical categorisations for asthma [16].

### Covariates

Baseline information included demographic, disease and general health characteristics. A valid blood eosinophil count was the most recently recorded value in the 2 years prior to the index date, based on simplicity of use for primary care clinicians. For the primary analysis, eosinophil values within 2 weeks of an exacerbation, pneumonia episode or elevated C-reactive protein (>100 mg·L^−1^) were excluded as these would not reflect baseline state. An eosinophil threshold of <150 cells·µL^−1^ was used to categorise patients into the low-eosinophil group and ≥150 cells·µL^−1^ for the high-eosinophil group. This primary threshold was chosen in response to unpublished work at the time of study set-up [17], but with multiple alternative secondary thresholds assessed.

### Outcomes

The primary outcome was time to first exacerbation following the index date, which was selected as the outcome of most relevance to patients [18]. Exacerbations were defined as any of the following: code for COPD exacerbation; code for lower respiratory tract infection; prescription of exacerbation-specific antibiotic, *e.g.* amoxicillin/macrolides/doxycycline and oral steroid for 5–14 days; symptom of exacerbation (cough, breathlessness or sputum) plus prescription of exacerbation-specific antibiotic or oral steroid; hospital admission with COPD or acute respiratory cause as the primary cause of hospitalisation; or a COPD exacerbation code within a hospitalisation episode. Exacerbation events defined by prescriptions alone and occurring on the same date as spirometry, or a code implying rescue-pack administration, were excluded, as this suggested a visit for annual COPD review with provision of standby medication, rather than an exacerbation.

Secondary outcomes analysed were pneumonia episodes, and hospitalisations and death due to COPD, pneumonia or any cause (all time-to-event following index date). A pneumonia episode was defined as a CPRD code for pneumonia, hospital admission with an International Classification of Diseases (ICD)-10 pneumonia code, or a death certificate with pneumonia listed as a cause.

All outcome events occurring within 2 weeks of a previous episode, were counted as the same event. Events within 30 days of the index date were excluded to reduce protopathic bias, as in other studies [14].

### Missing data

Handling of missing data is detailed in the supplementary material.

### Statistical analysis

Stata (release SE13 64-bit) was used for all analysis. Data are presented as mean±sd, median (interquartile range (IQR)) or hazard ratios (HR) with 95% confidence intervals. Cox proportional hazards models assessed disease outcomes after index date, by drug group (ICS *versus* non-ICS). Inclusion of an interaction term looked for effect modification by blood eosinophils (due to the difference in response to treatment between eosinophil groups being more relevant than the effect size itself). Covariates were adjusted for if significant (p<0.10) in univariate Cox analysis, to reduce confounding by indication in terms of ICS *versus* non-ICS group.

Pre-specified subgroup analyses by baseline exacerbation frequency, ICS dose and history of asthma were performed (and by smoking status *post hoc*). A history of frequent exacerbations was defined as two or more in the year prior to the index date and less-frequent exacerbations defined as fewer than two in the year prior to the index date (exacerbations included hospitalisations for exacerbations, as defined earlier). Multiple sensitivity analyses explored whether different ways of defining the population (*e.g.* inclusion/exclusion of those with asthma and atopy) and alternative methods for handling medication adherence and outcome timing had any impact on overall results (full details in supplementary material).

## Results

### Characteristics of included population

There were 30 378 eligible patients, of whom 18 235 (60.0%) had a valid eosinophil count in primary care records. A further 8760 met exclusion criteria, leaving 9475 patients for analysis (supplementary figure S1).

**FIGURE 1:**
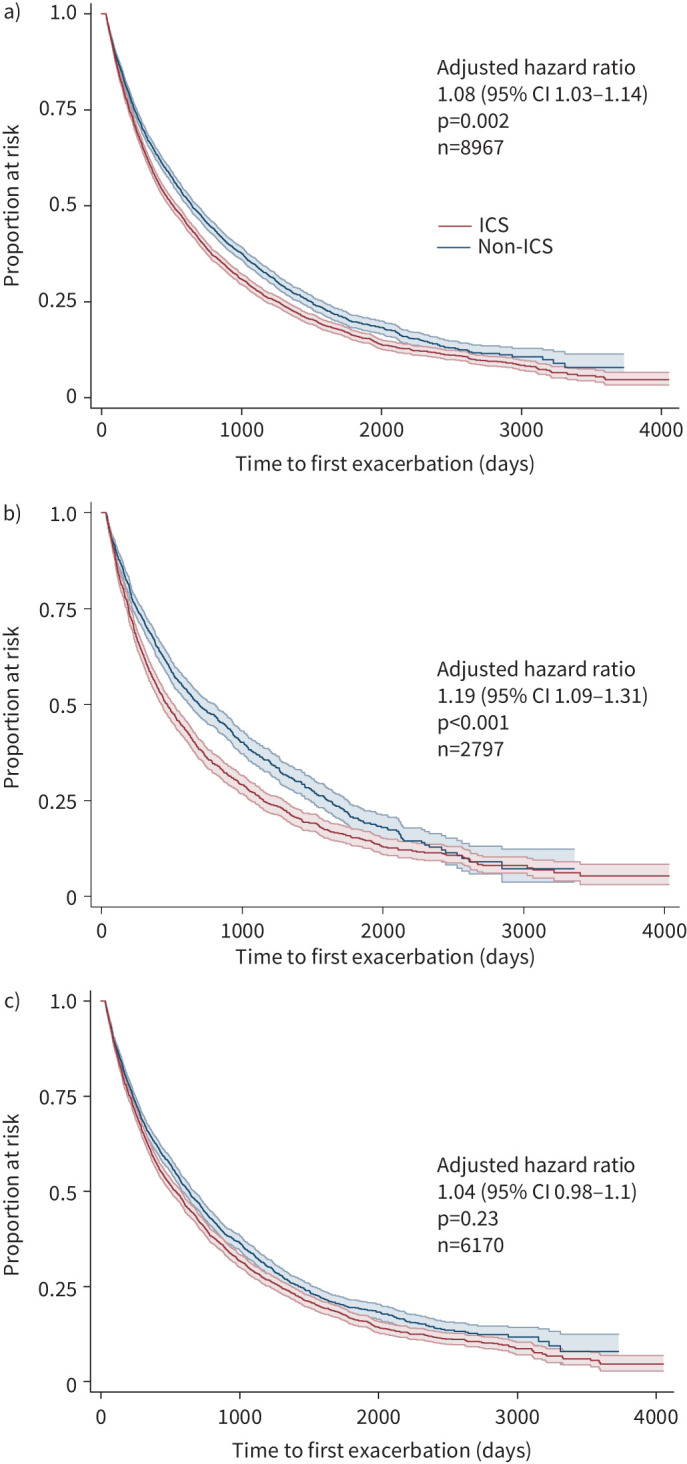
Kaplan–Meier curves for time to first exacerbation in inhaled corticosteroid (ICS) (red) *versus* non-ICS (blue) groups, a) overall and b, c) stratified by baseline blood eosinophil group (95% CI shaded). a) Whole group; b) low-eosinophil group (<150 cells·µL^−1^); c) high-eosinophil group (≥150 cells·µL^−1^). The interaction between the two eosinophil groups was significant (interaction of eosinophil group with treatment group 0.87, 95% CI 0.78–0.97; p=0.01, 15% absolute difference). Hazard ratios are from Cox regression including the interaction term and adjusted for covariates as follows: age category, sex, smoking status, year of index prescription, socioeconomic status, history of atopy, history of asthma, exacerbations in previous year, pneumonia episodes in previous year, oral steroid prescriptions in previous year, salbutamol inhaler prescriptions in previous year, theophylline in previous 2 years, oxygen use ever, nebulised therapies in previous 2 years, nonelective hospitalisations in previous year, general practitioner consultations in previous year, Charlson comorbidity index [19], influenza vaccination in previous year, pneumococcal vaccination in previous 5 years.

Table 1 shows baseline characteristics and distribution of patients between ICS and non-ICS treatment groups. Patients were more likely to be prescribed ICS therapy if they were younger, female, had previous asthma, more severe airflow limitation, or with a higher baseline exacerbation frequency, oral steroid use or hospital admissions (supplementary table S1). There were 4371 (46%) patients in the non-ICS group (prescribed LABA 19%, LAMA 77%, LAMA/LABA 4%) and 5104 in the ICS group (prescribed ICS 36%, ICS/LABA 62%, ICS/LAMA 3%). Prescriptions for ICS decreased by 81.6% over the decade of the study, whereas non-ICS prescriptions remained constant. A high eosinophil count (≥150 cells·µL^−1^) occurred in 69.0% of patients. There was no difference in treatment distribution between the ICS and non-ICS groups by eosinophil group (p=0.71).

**TABLE 1:** Distribution of patients between inhaled corticosteroid (ICS) and non-ICS groups by baseline characteristics

	**Overall**	**Non-ICS group**	**ICS group**
**Patients**	9475	4371	5104
**Age, years**	69.7±10.0	70.0±9.7	69.4±10.2
**Female**	4111 (43.4)	1809 (41.4)	2302 (45.1)
**Current smoker^#^**	3946 (41.8)	1836 (42.1)	2110 (41.6)
**Airflow limitation severity (most recent FEV_1_% predicted)^¶^**			
Mild (≥80%)	838 (11.9)	401 (11.3)	437 (12.5)
Moderate (50–80%)	3878 (55.0)	2110 (59.4)	1768 (50.6)
Severe (30–50%)	2010 (28.5)	914 (25.7)	1096 (31.4)
Very severe (<30%)	322 (4.6)	127 (3.6)	195 (5.6)
**Asthma >2 years previously**	1098 (11.6)	269 (6.2)	829 (16.2)
**History of atopy^+^**	2493 (26.3)	1107 (25.3)	1386 (27.2)
**Exacerbations in previous year**			
0	4887 (51.6)	2433 (55.7)	2454 (48.1)
1	2829 (29.9)	1250 (28.6)	1579 (30.9)
2	1165 (12.3)	466 (10.7)	699 (13.7)
⩾3	594 (6.3)	222 (5.1)	372 (7.3)
**Pneumonia episodes in previous year**			
0	7484 (79.0)	3514 (80.4)	3970 (77.8)
1	1500 (15.8)	660 (15.1)	840 (16.5)
⩾2	491 (5.2)	197 (4.5)	294 (5.8)
**Theophylline in previous 2** **years**	97 (1.0)	17 (0.4)	80 (1.6)
**Oxygen use ever**	46 (0.5)	19 (0.4)	27 (0.5)
**Nebulisers in previous 2** **years**	157 (1.7)	48 (1.1)	109 (2.1)
**Nonelective hospitalisations^§^ in previous year**			
0	7767 (82.0)	3663 (83.8)	4104 (80.4)
1	1277 (13.5)	529 (12.1)	748 (14.7)
⩾2	431 (4.6)	179 (4.1)	252 (4.9)
**GP consultations in previous year**			
0–3	2699 (28.5)	1280 (29.3)	1419 (27.8)
4–7	3381 (35.7)	1586 (36.3)	1795 (35.2)
⩾8	3395 (35.8)	1505 (34.4)	1890 (37.0)
**Influenza vaccination in previous year**	6710 (70.8)	3106 (71.1)	3604 (70.6)
**Blood eosinophil count (cells·µL^−1^)**			
Geometric mean	200	200	201
Median (IQR)	200 (100–300)	200 (100–300)	200 (100–300)

Data are presented as n, mean±sd or n (%), unless otherwise stated. Percentages are column percentages. FEV_1_: forced expiratory volume in 1 s; GP: general practitioner; IQR: interquartile range. ^#^: n=9442 for smoking status (only past or current smokers included); ^¶^: n=7048 for airflow limitation severity; ^+^: atopy defined using presence of codes for allergy, eczema or hay fever; ^§^: for any cause.

### Primary analysis

468 patients experienced an exacerbation in the first month after initiating treatment (58.1% in the ICS group) and were excluded from the primary analysis. The remaining 9007 patients provided 38 421 years of follow-up (median 3.8 years per patient, IQR 2.1–6.0 years; range 0.5–11.1 years); 6478 (71.9%) of them experienced an exacerbation during follow-up. The median (95% CI) time to first exacerbation was 645 (95% CI 615–686) days in the non-ICS group and 512 (483–541) days in the ICS group (unadjusted HR ICS *versus* non-ICS 1.17, 95% CI 1.12–1.23, p<0.001; adjusted HR 1.08, 95% CI 1.03–1.14, p=0.002; figure 1a). Following stratification for baseline eosinophils, the adjusted HR was 1.19 (95% CI 1.09–1.31; p<0.001; figure 1b) in the low-eosinophils group and 1.04 (95% CI 0.98–1.10, p=0.23; figure 1c) in the high-eosinophils group (15% absolute difference; interaction of eosinophil group with treatment group 0.87, 95% CI 0.78–0.97, p=0.01; figure 1).

### Subgroup and sensitivity analyses of primary analysis

Risk of exacerbations on ICS was lower in those with high eosinophils and history of frequent exacerbations compared to those with low eosinophils and less-frequent exacerbations (HR 0.94, 95% CI 0.82–1.07, p=0.34 *versus* 1.21, 1.10–1.34, p=0.001; table 2). Multiple sensitivity analyses including using the eosinophil count average of the most recent two, three or all eosinophil counts instead of the most recent value, excluding the highest values (≥500 cells·µL^−1^), and including eosinophil values close to acute events, made no difference to overall results (supplementary table S2).

**TABLE 2:** Subgroup analysis of inhaled corticosteroid (ICS) *versus* non-ICS treatment, stratified by blood eosinophil group and by baseline exacerbation frequency

	**Low-eosinophil group** **(<150 cells·µL^−1^)**	**High-eosinophil group** **(≥150 cells·µL^−1^)**	**Interaction HR and p-value**
**Low exacerbation rate (0 or 1)**	1.21 (1.10–1.34) p=0.001 n=2299	1.07 (1.00–1.15) p=0.06 n=5068	0.88 (0.78–0.99) p=0.04
**Higher exacerbation rate (≥2)**	1.18 (0.97–1.44) p=0.11 n=498	0.94 (0.82–1.07) p=0.34 n=1102	0.79 (0.62–1.00) p=0.06

Data are presented as hazard ratio (HR) (95% CI), unless otherwise stated. HRs are for time to first exacerbation after treatment initiation, for ICS *versus* non-ICS treatment. HRs are from Cox regression including the interaction term and adjusted for covariates, as listed in figure 1. n=9007.

### Analysis with different eosinophil thresholds

**FIGURE 2:**
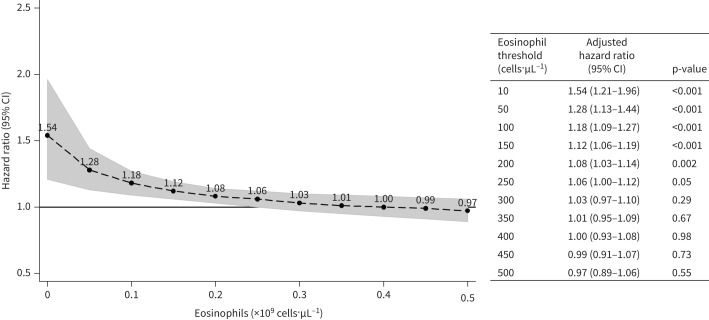
Hazard ratios for time to first exacerbation for inhaled corticosteroid (ICS) *versus* non-ICS treatment, at different eosinophil counts. Hazard ratios are from Cox regression including the interaction term and adjusted for covariates as detailed in figure 1, but with eosinophils in the model as a continuous variable (logarithmically transformed). The interaction of eosinophils with ICS treatment group was significant in this model (p=0.004). Deviation of the association from log-linearity was assessed by a likelihood ratio test comparing models with categorical eosinophils (p=0.23). The shaded area shows 95% confidence intervals.

Using a threshold of 340 cells·µL^−1^ [20, 21] instead of 150 cells·µL^−1^, the number of patients in the high-eosinophil group decreased from 69.0% to 19.4% (supplementary table S3). Decreasing HR for ICS treatment as eosinophil count increased was found with increasing eosinophil thresholds, categories, and in continuous analysis (supplementary table S4), and ICS only reduced exacerbations at much higher eosinophil counts (≥450 cells·µL^−1^) (figure 2).

**TABLE 3:** Pneumonia outcomes stratified by baseline blood eosinophil group

	**Low-eosinophil group** **(<150 cells·µL^−1^)**	**High-eosinophil group** **(≥150 cells·µL^−1^)**	**Interaction HR and p*-*value**
**Pneumonia episodes**	1.10 (0.99–1.24) p=0.09 n=2832	1.05 (0.97–1.13) p=0.24 n=6321	0.95 (0.83–1.08) p=0.44
**Hospitalisation due to pneumonia**	1.26 (1.05–1.50) p=0.01 n=2910	1.00 (0.88–1.14) p>0.99 n=6499	0.80 (0.64–0.99) p=0.04
**Death due to pneumonia**	1.19 (0.50–2.84) p=0.70 n=2918	0.53 (0.27–1.05) p=0.07 n=6517	0.44 (0.65–4.42) p=0.14

Data are presented as hazard ratio (HR) (95% CI), unless otherwise stated. HRs are for time to first event after treatment initiation, for inhaled corticosteroid (ICS) *versus* non-ICS treatment. HRs are from Cox regression including interaction term and adjusted for covariates as detailed in figure 1.

### Secondary outcomes

At eosinophil levels <150 cells·µL^−1^, ICS use was associated with pneumonia, with significant interaction in pneumonia hospitalisations (HR in low-eosinophil group 1.26, 95% CI 1.05–1.50; p*-*value for interaction p=0.04) (table 3). Time-to-event analyses for different eosinophil thresholds for the pre-specified secondary outcomes are presented in supplementary table S5.

## Discussion

### Summary of main findings

In this real-world study comparing ICS with non-ICS treatment in patients with COPD, there was a statistically significant interaction between ICS treatment and baseline blood eosinophil count. This translated as a 15% lower absolute risk of subsequent exacerbations in patients with higher baseline eosinophil counts who were prescribed an ICS treatment, compared with patients with lower eosinophils who were prescribed ICS. In patients prescribed an ICS there was an eosinophil dose-response with risk of subsequent exacerbation greatest in those with lower eosinophil counts (*i.e.* <150 cells·µL^−1^). Results were unchanged when mean of eosinophil counts was used instead of most recent values, or when those taken close to an acute illness were included. In secondary analyses, there was a higher risk of pneumonia hospitalisation in patients receiving ICS treatment with eosinophil counts <150 cells·µL^−1^.

Contrary to national and international COPD guidelines [22, 23], almost one in five patients were initiated on ICS monotherapy for their COPD, but this has been replicated in other real-life database studies [24, 25]. We found that those prescribed ICS had more “asthma-like” features (*e.g.* younger, female, previous asthma or oral steroid use), which could suggest that patients with ongoing asthma might be included in the cohort. However, these patients had been coded as COPD and had spirometry diagnostic of COPD, and sensitivity analyses excluding or including all patients with asthma did not change results.

### Strengths and weaknesses

We included a large number of patients exposed to routine primary care rather than a highly selected trial population [8]. Other strengths include the new-user cohort study design which avoids immortal time bias that may be present in pharmaco-epidemiological studies [26]; evaluation of steroid-naïve patients, as ICS treatment can affect blood eosinophil values [13]; and use of key variables of interest such as blood results and prescriptions which are generally inputted automatically and so should have virtually complete coverage and accuracy. In this study, missing information is likely to be equally distributed between the eosinophil and treatment groups and should therefore not impact findings.

There is a risk of residual confounding by indication, *i.e.* there may be unmeasured differences between treatment groups which have not been accounted for. This may partly explain the worse outcome seen with ICS treatment in this study compared to in trials, which have in general found either no or a small benefit of ICS on exacerbation outcomes [2, 27–29], including “real-life” trials in primary care, such as the Salford Lung Study [30]. However, it is the difference in treatment effect size between eosinophil groups, rather than the absolute values, which are important for assessing the role of eosinophils in predicting ICS responsiveness. Importantly, there was no difference in treatment distribution between the ICS and non-ICS groups by eosinophil group.

Our choice of primary eosinophil count threshold of 150 cells·µL^−1^ is lower than in other studies and is not the threshold for considering initiation in global guidelines [1]. However, this was a pre-specified cut-off based on data available during protocol development. Our observed eosinophil–treatment association was consistent across repeated different thresholds in addition to methodological sensitivity analyses, while a dose–response relationship was seen using continuous analysis of eosinophils.

Our sample size was reduced by almost half due to discontinuation of the new inhaled treatment within 6 months. This may be because of a conscious trialling of medication, a change to an alternative or the patient failing to request prescriptions. However, sensitivity analysis using the full intention-to-treat population, as well as complete on-treatment analysis, made minimal difference to results. Other sensitivity analyses including inclusion of those with an outcome in the first month, and inclusion of covariates with large amounts of missing data, made minimal difference to overall findings.

### Comparison with other studies

A systematic review and meta-analysis [5] including 11 *post hoc* analyses of randomised controlled trials (RCTs), and five retrospective observational studies, found a relative risk of ICS on reducing exacerbation risk of 0.65 (0.52–0.79) with eosinophil counts >150 cells·µL^−1^ and 0.87 (0.79–0.95) with eosinophil counts <150 cells·µL^−1^ (four studies used this threshold), and a dose-response of increasing benefit with increasing eosinophil count. However, four out of the five observational studies showed no association. Similar findings have been found in continuous eosinophil analysis of previous trials, except that the benefit of ICS was seen at lower eosinophil counts of 100 cells·µL^−1^ [31] and 180 cells·µL^−1^ [32]. Non-ICS treatment appears more favourable in the lowest eosinophil groups [33]. These differences in findings between observational studies and RCTs may be explained by patients who are more unwell being commenced on ICS *versus* non-ICS treatment, and indeed this was confirmed in the differences between the two groups at baseline, which may relate to other unknown confounders.

Two other studies also used CPRD data to address the same objective. Oshagbemi *et al.* [34] found a similar HR of exacerbations in patients prescribed ICS *versus* non-ICS treatment, but that stratification of ICS use by either absolute or relative eosinophil counts did not identify significant differences in risk. However, they excluded all patients with asthma and those who had had any exacerbations in the baseline period. Suissa *et al*. [35] was a new-user cohort study directly comparing LABA/ICS with LAMA, which found a slightly lower HR for exacerbations than in our study (0.95, 95% CI 0.90–1.01 *versus* 1.08, 95% CI 1.03–1.14), but a higher HR for pneumonia risk (HR 1.37, 95% CI 1.17–1.60 *versus* 1.06, 95% CI 1.00–1.13). Despite a number of differences in the methods between our studies, they found a similar association, but with the benefit of ICS seen at lower eosinophil counts.

Our secondary outcomes investigated the relationship of pneumonia events, including severe hospitalised events and pneumonia mortality. We confirm a higher risk of pneumonia in patients with COPD receiving ICS therapy with eosinophils <150 cells·µL^−1^. This risk was greatest and significant in severe pneumonia events. These findings have been demonstrated before [35, 36], and may relate to an increased bacterial load in ICS-treated patients who have lower (≤2%) blood eosinophils [37].

### Application

Our study findings suggest that eosinophil count can be used to predict risk–benefit of ICS treatment: at lower eosinophil levels (especially <150 cells·µL^−1^) there is a much higher risk of exacerbations and pneumonia hospitalisation with ICS treatment. In this cohort, benefit of ICS treatment was only seen in those with baseline eosinophil counts ≥450 cells·µL^−1^, and suggests that ICS treatment should particularly be avoided in those with low eosinophil counts. This fits with current Global Initiative for Chronic Obstructive Lung Disease recommendations [38] and National Institute for Health and Care Excellence guidelines [39].

It is not clear whether one or more eosinophil count estimation is required to guide ICS initiation in patients with COPD in clinical practice where often many results are available [40, 41]. Our sensitivity analyses demonstrated that decisions to initiate ICS could be made irrespective of whether the last recorded eosinophil value or an average of multiple results were used.

### Conclusions

We recommend considering a more limited approach to ICS prescribing and advise against ICS treatment initiation at low blood eosinophil levels (<150 cells·µL^−1^), where there is a lower likelihood of treatment benefit and potential harms.

## Supplementary material

10.1183/23120541.00606-2021.Supp1 **Please note:** supplementary material is not edited by the Editorial Office, and is uploaded as it has been supplied by the author.
Supplementary material 00606-2021.SUPPLEMENT
