# Supplementary material for: Blood eosinophils to guide inhaled maintenance therapy in a primary care COPD population
Source: ERJ Open Res. 2021 Feb 7;8(1):00606-2021. doi: 10.1183/23120541.00606-2021 (PMC8819252; doi:10.1183/23120541.00606-2021)
Supplement: Supplementary file 1 [file 00606-2021.SUPPLEMENT.pdf]

**Online data supplement**

**Contents**

Supplementary Methods: Further details of methods
Supplementary Table 1: Logistic regression for distribution of patients between ICS and non-ICS groups by baseline characteristics
Supplementary Table 2: Sensitivity and subgroup analyses for time-to-first exacerbation ICS vs. non-ICS and interaction with blood eosinophil count, in overall population (both eosinophil groups)
Supplementary Table 3: Distribution of patients between ICS and non-ICS groups by different blood eosinophil thresholds
Supplementary Table 4: Outcomes and interactions for different eosinophil thresholds
Supplementary Table 5: Secondary outcomes
Supplementary Figure 1: Study flow chart for inclusion of patients
Supplementary References: Online data supplement references

**Supplementary Methods: Further details of methods**

**Data source**
In the UK, 98% of the population are registered with a National Health Service (NHS) general practitioner (GP). GPs are the primary contact for the majority of health-related issues, and the gatekeepers for accessing secondary care, with the majority of COPD management taking place in primary care. Information is recorded routinely on computers using a coding system combined with free text, and using a unique NHS number, which remains with the patient if they move GPs [1].

The CPRD is a primary care database of anonymised medical records from GPs, with 14.5 million patients included (CPRD August 2016 release). Patients in the CPRD are broadly representative of the UK general population in terms of age, sex and ethnicity. GPs are the gatekeepers of primary care and specialist referrals in the UK. The CPRD is therefore a rich source of health data for research, including data on demographics, symptoms, tests, diagnoses and therapies prescribed [1]. Approximately half of the data is linked with other datasets: in this study we obtained linkage with Hospital Episode Statistics (HES) which gives information on hospitalisations and diagnoses, Index of Multiple Deprivation (IMD) (deprivation score) and Office for National Statistics data on causes of death.

For all code lists used to determine diagnoses, therapies or tests, we used search terms combined with QOF code lists, which were then independently selected by two clinicians (HFA and DMcC) and any disagreements discussed and adjudicated by a third clinician (MB). We used previously validated code lists where available [2, 3].

**Exposure definition**
In the UK, blood eosinophil count is provided automatically as part of a request for a full blood count. Blood eosinophil readings were transformed from other units or percentage values to cells/µL. Values of zero or ≥1500 cells/µL, or where the total white cell count was outside of the range 3-15 $x10^9$/L, were excluded, as they were felt more likely to be a data error (missing values may be entered as zero), or a haematopoietic problem and not truly representative of baseline state. We also calculated season of eosinophil test in case of variation of values throughout the year.

**Sensitivity and subgroup analyses**
We planned sensitivity analyses as follows: different thresholds for blood eosinophil counts (100, 200, 300, 340 (post-hoc) [4, 5], 400 and 500 cells/µL, percentage eosinophils (<2%, ≥2-<4% and ≥4%)) and continuously (which tells us if there is a linear effect for presence or absence of association which is most useful to look at for overall association; log-transformed data were used as eosinophils are non-normally distributed); using mean of blood eosinophils over prior two years, rather than most recent value before index date; including patients with currently active asthma (coded in the last two years); excluding patients with any history of asthma (coded ever); excluding patients with a history of atopy; including blood eosinophil values close to an acute event (exacerbation/pneumonia episode or raised CRP); and including those who experienced an event in the first month after index date. Post-hoc sensitivity analyses mainly responded to unforeseen issues with the data: including those who remained on their index medication for less than 6 months; censoring

by duration of index medication; censoring by time to initiation of a new drug from the alternative drug class (i.e. change of category ICS to non-ICS or vice versa); censoring by duration of medication and time to initiation of new drug (whichever occurred earlier); including season of blood test in the model; excluding those with the highest eosinophils (≥500 cells/µL); including airflow limitation severity and MRC breathlessness scale in the model; and using mean of the most recent two or three eosinophil counts rather than the single most recent.

The main subgroup analysis was by baseline exacerbation frequency, and we also planned stratification by ICS dose. Following recent publication of post-hoc analysis of trials suggesting that current smokers particularly benefit from ICS [6], we conducted a post-hoc subgroup analysis by smoking status. We also conducted post-hoc analysis stratifying eosinophils into low (<150), medium (150-<340) and high (≥340) groups.

**Missing data**

For the assessment of clinical diagnosis and outcomes, we assumed that absence of any relevant medical code meant true absence of disease. We expected age, sex and prescriptions to be well recorded in the cohort and so planned a complete case analysis. Spirometry was poorly coded and so we used standard formulae [7] to calculate percentage predicted $FEV_1$ from data available. Where height was missing, we used the mean height of that sex and 10-year age category in the cohort. Nonetheless, $FEV_1$ percentage predicted remained missing for a quarter of the population and therefore we did not include this in the main analysis, but conducted a sensitivity analysis to assess the effect of incorporating it into the model. The same was true for MRC breathlessness score, which was missing for approximately half of patients. We did not perform multiple imputation because the assumption that the missing data were missing completely at random or missing at random may not have been realistic [8], indeed in early analyses there were significant differences between groups. It was not possible due to limitations in what had been coded to confirm whether spirometry was pre- or post-bronchodilator.

**Supplementary Table 1: Logistic regression for distribution of patients between ICS and non-ICS groups by baseline characteristics**

| **Baseline variable n=9,475** | **Unadjusted odds ratio for ICS vs. non-ICS group (95% CI, P Value)** | **Adjusted odds ratio for ICS vs. non-ICS group[a] (95% CI, P Value)** |
|---|---|---|
| Age group in years | | |
| 40-49 | 1.92 (1.47-2.50) P<*0.*001 | 1.92 (1.45-2.55) P<0.001 |
| 50-59 | 1.14 (1.00-1.30) P=0.06 | 1.10 (0.95-1.27) P=0.20 |
| 60-69 | 1.10 (1.00-1.22) P=0.06 | 1.14 (1.03-1.27) P=0.01 |
| 70-79 (ref) | | |
| 80-89 | 1.16 (1.03-1.32) P=0.01 | 1.17 (1.03-1.33) P=0.02 |
| >=90 | 0.92 (0.60-1.39) P=0.06 | 0.90 (0.58-1.41) P=0.66 |
| Female | 1.16 (1.07-1.26) P<0.001 | 1.11 (1.02-1.21) P=0.02 |
| Current smoker[b] | 0.98 (0.90-1.06) P=0.61 | |
| History of atopy | 1.10 (1.00-1.20) P=0.04 | 1.04 (0.95-1.15) P=0.40 |
| Asthma >2 years previously | 2.96 (2.56-3.42) P<0.001 | 2.64 (2.27-3.07) P<0.001 |
| Airflow limitation severity (most recent $FEV_1$ % predicted)[c] | | |
| Mild (≥80%) (ref) | | |
| Moderate (50-80%) | 0.77 (0.66-0.89) P=0.001 | |
| Severe (30-50%) | 1.10 (0.94-1.30) P=0.25 | |
| Very severe (<30%) | 1.41 (1.08-1.83) P=0.01 | |
| MRC breathlessness scale[c] | | |
| 1 (least severe) (ref) | | |
| 2 | 0.70 (0.58-0.85) P<0.001 | |
| 3 | 0.69 (0.57-0.84) P<0.001 | |
| 4 | 0.89 (0.71-1.13) P=0.34 | |
| 5 (most severe) | 1.12 (0.70-1.78) P=0.64 | |
| Exacerbations in previous year | | |
| 0 (ref) | | |
| 1 | 1.25 (1.14-1.37) P<0.001 | 1.22 (1.10-1.37) P<0.001 |
| 2 | 1.49 (1.31-1.69) P<0.001 | 1.46 (1.24-1.72) P<0.001 |
| 3 or more | 1.66 (1.39-1.98) P<0.001 | 1.51 (1.20-1.90) P<0.001 |
| Pneumonia episodes in previous year | | |
| 0 (ref) | | |
| 1 | 1.13 (1.00-1.26) P=0.04 | 0.89 (0.77-1.01) P=0.08 |
| 2 or more | 1.32 (1.10-1.59) P=0.003 | 0.85 (0.67-1.07) P=0.17 |
| Oral steroids in previous year | | |
| 0 (ref) | | |
| 1 | 1.48 (1.32-1.65) P<0.001 | 1.39 (1.22-1.57) P<0.001 |
| 2 | 1.76 (1.45-2.13) P<0.001 | 1.55 (1.25-1.91) P<0.001 |
| Salbutamol inhalers in previous year | | |
| 0 (ref) | | |
| 1 | 0.90 (0.80-1.01) P=0.08 | 0.88 (0.78-0.99) P=0.04 |
| 2 | 1.05 (0.91-1.22) P=0.50 | 0.91 (0.78-1.07) P=0.26 |
| 3-5 | 1.18 (0.95-1.22) P=0.26 | 0.89 (0.78-1.02) P=0.09 |
| 6 or more | 1.25 (1.12-1.40) P<0.001 | 0.95 (0.84-1.07) P=0.36 |
| Theophylline in two previous years | 4.08 (2.41-6.89) P<0.001 | 2.61 (1.51-4.53) P=0.001 |
| Oxygen use ever | 1.22 (0.68-2.19) P=0.51 | |
| Nebulisers in two previous years | 1.97 (1.40-2.77) P<0.001 | 1.25 (0.87-1.81) P=0.23 |
| Charlson comorbidity index[d] | | |
| 0 (ref) | | |
| 1 | 0.96 (0.86-1.08) P=0.50 | 0.96 (0.85-1.08) P=0.49 |
| 2 or more | 0.83 (0.76-0.91) P<0.001 | 0.90 (0.81-1.00) P=0.05 |

| **Supplementary Table 1 (continued)** | | |
|---|---|---|
| **Baseline variable n=9,475** | **Unadjusted odds ratio for ICS vs. non-ICS group (95% CI, P Value)** | **Adjusted odds ratio for ICS vs. non-ICS group[a] (95% CI, P Value)** |
| Non-elective hospitalisations in previous year | | |
| 0 (ref) | | |
| 1 | 1.26 (1.12-1.42) P<0.001 | 1.20 (1.05-1.36) P=0.006 |
| 2 or more | 1.26 (1.03-1.53) P=0.02 | 1.20 (0.97-1.48) P=0.09 |
| GP consultations in previous year | | |
| 0-3 (ref) | | |
| 4-7 | 1.02 (0.92-1.13) P=0.69 | 0.97 (0.87-1.08) P=0.54 |
| 8 or more | 1.13 (1.02-1.25) P=0.02 | 1.01 (0.90-1.12) P=0.91 |
| Influenza vaccination in previous year | 0.98 (0.90-1.07) P=0.63 | |
| Pneumococcal vaccination in previous 5 years | 1.12 (1.03-1.22) P=0.007 | 0.96 (0.87-1.05) P=.37 |

[a] Odds ratio calculated using logistic regression. Adjusted odds ratios include baseline variables significant *P*<0.10 in univariate analysis.
[b] n=9,442 for smoking status; reference group was ex-smokers.
[c] Due to large amounts of missing data for airflow limitation severity (n=7,048) and MRC breathlessness score (n=4,272) these were not included in the multivariate analysis.
[d] Charlson comorbidity index gives categories of comorbid disease and provides a summary of disease burden for individual patients [9].

**Supplementary Table 2: Sensitivity and subgroup analyses for time-to-first exacerbation ICS vs. non-ICS and interaction with blood eosinophil count**

| Groups as applicable | 150 cells/µL eosinophil threshold | | 340 cells/µL eosinophil threshold | | Continuous eosinophils[a] |
|---|---|---|---|---|---|
| | Hazard ratio in low group[b] | Interaction[c] | Hazard ratio in low group[b] | Interaction[c] | Interaction[c] |
| **Main** | | | | | |
| (n=9,007) | 1.19 (1.09-1.31) P<0.001 | 0.87 (0.78-0.97) P=0.01 | 1.09 (1.03-1.16) P=0.002 | 0.95 (0.84-1.08) P =0.43 | 0.89 (0.82-0.96) P=0.004 |
| **Smoking status (post-hoc subgroup analysis)** | | | | | |
| Ex-smokers (n=5,261) | 1.15 (1.02-1.30) P=0.02 | 0.91 (0.79-1.05) P=0.22 | 1.09 (1.01-1.18) P=0.02 | 0.95 (0.80-1.12) P=0.52 | 0.92 (0.83-1.03) P=0.14 |
| Current smokers (n=3,779) | 1.24 (1.09-1.43) P=0.002 | 0.83 (0.70-0.97) P=0.02 | 1.10 (1.01-1.20) P=0.03 | 0.96 (0.79-1.18) P=0.73 | 0.85 (0.76-0.96) P=0.009 |
| **Asthma status (main analysis excludes asthma coded in previous two years but includes those with history of asthma)** | | | | | |
| Excluding any asthma (n=7,981) | 1.21 (1.10-1.33) P<.001 | 0.85 (0.76-0.96) P=0.006 | 1.09 (1.02-1.15) P=0.007 | 0.98 (0.85-1.12) P=0.74 | 0.88 (0.81-0.96) P=0.004 |
| Including active asthma (n=9,326) | 1.20 (1.10-1.31) P<.001 | 0.87 (0.78-0.96) P=0.008 | 1.10 (1.04-1.16) P=0.001 | 0.94 (0.83-1.06) P=0.31 | 0.88 (0.82-0.95) P=0.002 |
| **Atopy (main analysis includes those with atopy)** | | | | | |
| Excluding any atopy (n=6,648) | 1.19 (1.07-1.33) P=0.001 | 0.88 (0.78-1.00) P=0.04 | 1.09 (1.02-1.17) P=0.009 | 1.00 (0.86-1.16) P=0.98 | 0.92 (0.83-1.01) P=0.07 |
| **Dose of ICS (subgroup analysis)** | | | | | |
| ≤500µg BDP equivalent (n=5,921) | 1.14 (1.01-1.29) P=0.03 | 0.89 (0.77-1.03) P=0.11 | 1.09 (1.01-1.18) P=0.02 | 0.83 (0.70-0.99) P=0.03 | 0.86 (0.77-0.95) P=0.004 |
| 500-1000 µg BDP equivalent (n=5,552) | 1.22 (1.08-1.40) P=0.002 | 0.79 (0.68-0.93) P=0.003 | 1.04 (0.96-1.13) P=0.36 | 1.02 (0.85-1.23) P=0.80 | 0.90 (0.80-1.01) P=0.08 |
| >1000 µg BDP equivalent (n=5,095) | 1.29 (1.11-1.50) P=0.001 | 0.91 (0.77-1.09) P=0.31 | 1.20 (1.09-1.32) P<0.001 | 1.04 (0.85-1.28) P=0.69 | 0.92 (0.81-1.05) P=0.22 |
| **Including severity and MRC breathlessness scale (not included in main analysis due to large amounts of missing data)** | | | | | |
| Including severity and MRC (n=3,706) | 1.17 (1.01-1.36) P=0.04 | 0.85 (0.72-1.02) P=0.08 | 1.05 (0.96-1.16) P=0.29 | 1.00 (0.81-1.23) P=0.98 | 0.91 (0.79-1.04) P=0.15 |
| **Protopathic bias (main analysis excludes those with exacerbation in first month after treatment initiation)** | | | | | |
| Including outcome in first month (n=9,475) | 1.19 (1.09-1.30) P<0.001 | 0.87 (0.78-0.96) P=0.007 | 1.10 (1.04-1.16) P=0.001 | 0.92 (0.81-1.04) P=0.17 | 0.88 (0.81-0.95) P=0.001 |
| **Intention-to-treat (main analysis only includes those who stayed on their new medication for at least 6 months) (post-hoc)** | | | | | |
| Including <6m treatment duration (n=15,941) | 1.13 (1.05-1.21) P=0.001 | 0.91 (0.84-0.99) P=0.026 | 1.07 (1.02-1.18) P=0.003 | 0.93 (0.84-1.02) P=0.14 | 0.93 (0.87-0.99) P=0.02 |

| | | | | | |
|---|---|---|---|---|---|
| **Censoring by initiation of new drug in alternative treatment group (ICS or non-ICS) (post-hoc)** | | | | | |
| Censoring by time to new drug (n=9,007) | 1.31 (1.17-1.46) P<0.001 | 0.82 (0.72-0.93) P=0.002 | 1.17 (1.09-1.25) P<0.001 | 0.87 (0.75-1.01) P=0.07 | 0.85 (0.77-0.94) P=0.001 |
| **Censoring by duration of time on new medication (post-hoc)** | | | | | |
| Excluding <6m treatment duration (n=9,007) | 1.24 (1.12-1.37) P<0.001 | 0.87 (0.77-0.98) P=0.02 | 1.13 (1.06-1.21) P<0.001 | 0.97 (0.84-1.11) P=0.63 | 0.89 (0.82-0.97) P=0.01 |
| Including <6m treatment duration (n=15,941) | 1.23 (1.11-1.36) P<0.001 | 0.88 (0.79-0.99) P=0.04 | 1.14 (1.07-1.22) P<0.001 | 0.94 (0.82-1.07) P=0.35 | 0.89 (0.82-0.97) P=0.008 |
| **Censoring by initiation of new drug in alternative treatment group (ICS or non-ICS) or duration of time on new medication (earlier date where both apply) (post-hoc)** | | | | | |
| Excluding <6m treatment duration (n=9,007) | 1.33 (1.18-1.49) P<0.001 | 0.82 (0.72-0.94) P=0.005 | 1.19 (1.10-1.28) P<0.001 | 0.89 (0.76-1.05) P=0.17 | 0.86 (0.77-0.95) P=0.004 |
| Including <6m treatment duration (n=15,941) | 1.30 (1.16-1.46) P<0.001 | 0.85 (0.74-0.97) P=0.02 | 1.20 (1.11-1.28) P<0.001 | 0.87 (0.74-1.02) P=0.08 | 0.86 (0.78-0.95) P=0.003 |
| **Eosinophil means (main analysis uses most recent eosinophil result)** | | | | | |
| Using mean of all previous results (n=9,007) | 1.18 (1.07-1.30) P=0.001 | 0.89 (0.79-0.99) P=0.03 | 1.10 (1.04-1.16) P=0.002 | 0.94 (0.83-1.07) P=0.36 | 0.90 (0.83-0.98) P=0.01 |
| Using mean of last two results (n=9,007) | 1.20 (1.08-1.32) P<0.001 | 0.88 (0.78-0.98) P=0.02 | 1.10 (1.04-1.17) P=0.001 | 0.93 (0.83-1.05) P=0.25 | 0.90 (0.83-0.98) P=0.01 |
| Using mean of last three results (n=9,007) | 1.19 (1.08-1.31) P<0.001 | 0.88 (0.78-0.98) P=0.02 | 1.10 (1.03-1.16) P=0.002 | 0.95 (0.84-1.08) P=0.42 | 0.90 (0.82-0.97) P=0.009 |
| **Including season of eosinophil test as variable in model (post-hoc)** | | | | | |
| Including eosinophil test season (n=9,007) | 1.19 (1.09-1.30) P<0.001 | 0.87 (0.78-0.97) P=0.01 | 1.10 (1.03-1.16) P=0.002 | 0.95 (0.84-1.08) P=0.45 | 0.89 (0.82-0.96) P=0.004 |
| **Excluding those with eosinophils ≥500 cells/μL (post-hoc)** | | | | | |
| Excluding eosinophils ≥500 cells/μL | 1.18 (1.08-1.30) P<0.001 | 0.87 (0.78-0.97) P=0.01 | 1.09 (1.03-1.15) P=0.004 | 0.93 (0.79-1.09) P=0.41 | 0.86 (0.78-0.94) P=0.002 |
| **Including eosinophil values close to acute events (exacerbation/pneumonia/episode/C-reactive protein >100mg/L) which main analysis excludes** | | | | | |
| Including eosinophils close to acute event (n=9,007) | 1.18 (1.08-1.29) P<0.001 | 0.89 (0.80-0.99) P=0.03 | 1.10 (1.03-1.16) P=0.002 | 0.95 (0.84-1.08) P=0.46 | 0.90 (0.83-0.97) P=0.007 |

BDP, beclomethasone dipropionate estimated equivalent - [a] Continuous eosinophils were logarithmically transformed for analyses. [b] Hazard ratios are for time-to-first exacerbation comparing ICS with non-ICS treatment groups (hazard ratio >1 favours non-ICS treatment), in the low eosinophil group. Model is including the interaction term and adjusted for covariates as listed in Figure 1. Analyses are sensitivity analyses except where stated as subgroup analyses. [c] Interaction is the hazard ratio for the interaction of baseline blood eosinophils with treatment group, describing magnitude of difference (hazard ratio <1 describes reduced overall hazard ratio in ICS group, with higher eosinophils). Hazard ratio in the high eosinophil group can be calculated by multiplying the hazard ratio in the low group by the interaction term. 95% confidence intervals and P Values are given.

**Supplementary Table 3: Distribution of patients between ICS and non-ICS groups by different blood eosinophil thresholds**

| Eosinophil threshold (cells/µL) | Overall n=9,475 n (%) | Non-ICS group n=4,371 n (%) | ICS group n=5,104 n (%) | Unadjusted odds ratio ICS vs. non-ICS group (95% CI, P Value) | Adjusted odds ratio ICS vs. non-ICS group (95% CI, P Value) |
|---|---|---|---|---|---|
| ≥100 | 8,954 (94.5) | 4,140 (94.7) | 4,814 (94.3) | 0.93(0.78-1.11) P=0.40 | |
| ≥150 | 6,535 (69.0) | 3,023 (69.2) | 3,512 (68.8) | 0.98 (0.90-1.07) P=0.71 | |
| ≥200 | 5,924 (62.5) | 2,741 (62.7) | 3,183 (62.4) | 0.99 (0.91-1.07) P=0.73 | |
| ≥300 | 3,144 (33.2) | 1,438 (32.9) | 1,706 (33.4) | 1.02 (0.94-1.12) P=0.59 | |
| ≥340 | 1,842 (19.4) | 807 (18.5) | 1,035 (20.3) | 1.12 (1.01-1.24) P=0.03 | 1.15 (1.04-1.29) P=0.01 |
| ≥400 | 1,574 (16.6) | 687 (15.7) | 887 (17.4) | 1.13 (1.01-1.26) P=0.03 | 1.16 (1.04-1.31) P=0.01 |
| ≥500 | 815 (8.6) | 359 (8.2) | 456 (8.9) | 1.10 (0.95-1.27) P=0.21 | |
| Continuous (log scale) | | | | 1.02 (0.96-1.09) P=0.57 | |

Odds ratio calculated using logistic regression including baseline covariates significant P<0.10 in univariate analysis. Percentages are column percentages of those above the eosinophil threshold.

**Supplementary Table 4: Outcomes and interactions for different eosinophil thresholds and subgroups**

| | Hazard ratio for ICS vs non-ICS (95% confidence interval, P Value) | Interaction hazard ratio of eosinophils with treatment group (95% confidence interval, P Value) |
|---|---|---|
| **Eosinophil thresholds (sensitivity analysis)** | | |
| 100 cells/µL | 1.25 (1.00-1.55), P=0.05 | 0.86 (0.69-1.08), P=0.19 |
| 150 cells/µL (main analysis) | 1.19 (1.09-1.31), P<0.001 | 0.87 (0.78-0.97), P=0.01 |
| 200 cells/µL | 1.17 (1.08-1.27), P<0.001 | 0.88 (0.80-0.98), P=0.02 |
| 300 cells/µL | 1.12 (1.05-1.19), P<0.001 | 0.90 (0.81-1.01), P=0.06 |
| 340 cells/µL (post-hoc) | 1.09 (1.03-1.16), P=0.002 | 0.95 (0.84-1.08), P=0.43 |
| 400 cells/µL | 1.09 (1.03-1.15), P=0.002 | 0.96 (0.84-1.10), P=0.53 |
| 500 cells/µL | 1.08 (1.03-1.15), P=0.003 | 0.98 (0.82-1.18), P=0.83 |
| **Eosinophil categorical analysis (subgroup analysis)** | | |
| <150 cells/µL (n=2,819) | 1.19 (1.09-1.31), P<0.001 | 1.15 (1.02-1.29), P=0.01 |
| ≥150-<340 cells/µL* (n=4,451) | 1.04 (0.97-1.12) P=0.29 | |
| ≥340 cells/µL (n=1,737) | 1.04 (0.93-1.17) P=0.50 | 1.00 (0.88-1.15), P=0.98 |
| **Eosinophils as continuous variable (logarithmically transformed) (sensitivity analysis)** | | |
| Continuous | 1.18 (1.09-1.27), P<0.001 | 0.89 (0.82-0.96), P=0.004 |
| **Eosinophil percentages† (subgroup analysis)** | | |
| <2% (n=2,811) | 1.17 (1.07-1.28) P=0.001 | 1.08 (0.96-1.21), P=0.21 |
| 2-4% (n=3,795)* | 1.08 (1.00-1.17) P=0.04 | |
| ≥4% (n=2,388) | 1.00 (0.90-1.10) P=0.93 | 0.92 (0.81-1.04), P=0.18 |

* gives reference group for hazard ratios. † Eosinophil percentages are as percentage of total leucocytes; leucocytes missing for n=13. Proportional hazards assumption was valid for all eosinophil-related variables. Adjusted Cox regression model including interaction term as detailed in Figure 1 legend. Hazard ratios are for low eosinophil group for sensitivity analyses except for continuous eosinophils where this is set at 100 cells/µL; hazard ratio in the high eosinophil group can be calculated by multiplying the hazard ratio in the low group by the interaction term.

## Supplementary Table 5: Secondary outcomes

| **Number experiencing outcome/total§** | **150 cells/μL eosinophil threshold** | | **340 cells/μL eosinophil threshold** | | **Continuous eosinophils*** |
|---|---|---|---|---|---|
| | Hazard ratio† | Interaction‡ | Hazard ratio† | Interaction‡ | Interaction‡ |
| **Pneumonia episodes** | | | | | |
| n=4,210/9,192 | 1.10 (0.99-1.24) P=0.09 | 0.95 (0.83-1.08) P=0.44 | 1.06 (0.99-1.14) P=0.10 | 1.01 (0.87-1.19) P=0.86 | 0.99 (0.89-1.09) P=0.77 |
| **Hospitalisation due to any cause** | | | | | |
| n=6,392/9,007 | 1.04 (0.95-1.14) P=0.42 | 0.95 (0.85-1.06) P=0.35 | 1.01 (0.95-1.07) P=0.78 | 0.97 (0.86-1.10) P=0.67 | 0.96 (0.89-1.04) P=0.32 |
| **Hospitalisation due to pneumonia** | | | | | |
| n=1,533/9,449 | 1.26 (1.05-1.50) P=0.01 | 0.80 (0.64-0.99) P=0.04 | 1.13 (1.00-1.27) P=0.05 | 0.79 (0.61-1.03) P=0.08 | 0.88 (0.75-1.04) P=0.13 |
| **Hospitalisation due to COPD** | | | | | |
| n=2,621/9,384 | 1.17 (1.02-1.35) P=0.03 | 0.85 (0.72-1.01) P=0.07 | 1.05 (0.96-1.15) P=0.29 | 1.02 (0.83-1.25) P=0.85 | 0.92 (0.81-1.04) P=0.18 |
| **Death due to any cause** | | | | | |
| n=2,071/9,475 | 1.01 (0.87-1.19) P=0.86 | 0.93 (0.77-1.12) P=0.45 | 0.97 (0.87-1.07) P=0.52 | 0.99 (0.79-1.25) P=0.96 | 1.00 (0.87-1.15) P=0.96 |
| **Death due to pneumonia** | | | | | |
| n=61[ǁ] /9,475 | 1.19 (0.50-2.84) P=0.*70* | 0.44 (0.15-1.31) P=0.14 | 0.64 (0.35-1.17) P=0.15 | 1.74 (0.46-6.55) P=0.41 | 0.87 (0.38-1.99) P=0.75 |
| **Death due to COPD** | | | | | |
| n=568/9,475 | 1.07 (0.80-1.43) P=0.66 | 0.97 (0.68-1.39) P=0.87 | 1.04 (0.86-1.26) P=0.68 | 1.03 (0.66-1.62) P=0.90 | 1.06 (0.81-1.40) P=0.66 |

* Continuous eosinophils were logarithmically transformed for analyses. † Hazard ratios are for time-to-first event comparing ICS with non-ICS treatment groups (hazard ratio >1 favours non-ICS treatment), in the low eosinophil group. Model is including the interaction term and adjusted for covariates as listed in Figure 1 legend. ‡ Interaction is the hazard ratio for the interaction of baseline blood eosinophils with treatment group, describing magnitude of difference (hazard ratio <1 describes reduced overall hazard ratio in ICS group, with higher eosinophils). Hazard ratio in the high eosinophil group can be calculated by multiplying the hazard ratio in the low group by the interaction term. 95% confidence intervals and P Values are given. § As for exacerbations in main analysis, those experiencing the event of interest in the first month after initiating treatment were excluded. [ǁ] Low number of deaths due to pneumonia likely to be because of changes in coding of primary cause of death by the Office for National Statistics away from acute causes to chronic underlying causes (CPRD ONS Death Registration Data Data Specification V1.5 (15 August 2016).

## Supplementary Figure 1: Study flow chart for inclusion of patients

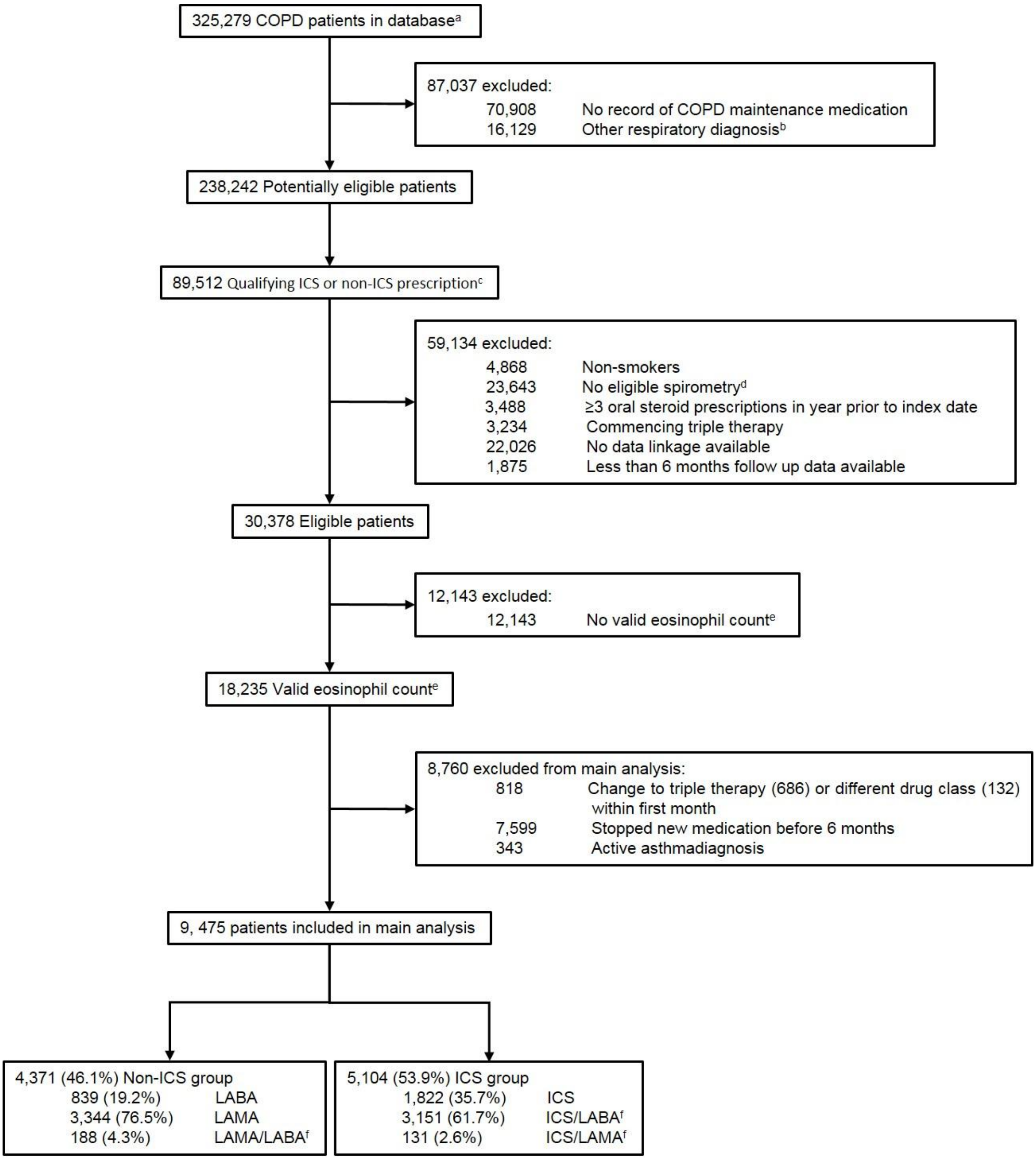


LAMA, long-acting muscarinic antagonist. LABA, long-acting $\beta_2$-agonist. ICS, inhaled corticosteroid

[a] CPRD August 2016 release.

[b] Other respiratory diagnoses excluded were bronchiectasis, cystic fibrosis and pulmonary fibrosis.

[c] Qualifying prescription required patients be ICS-naïve (no previous ICS in the preceding 12 months), have at least 2 years of data, 1st January 2005 or later, and be aged 40 or older on the date of the prescription, which was the first prescription for that drug in at least 12 months.

[d] Eligible spirometry was spirometry diagnostic for COPD ($FEV_1$/FVC ratio <0.7) at any time point.

[e] Valid eosinophil counts were those within the 2 years prior to the index date, with extreme values (zero or ≥1500 cells/µL) and those within 2 weeks of an acute event (exacerbation or pneumonia episode or C-reactive protein >100mg/L) excluded.

[f] Combination classes were either a single combined inhaler or separate inhalers with prescription issued on the same date.

## Supplementary References


1. Herrett E, Gallagher AM, Bhaskaran K, Forbes H, Mathur R, van Staa T, Smeeth L. Data Resource Profile: Clinical Practice Research Datalink (CPRD). *International journal of epidemiology* 2015: 44(3): 827-836.
2. Quint JK, Mullerova H, DiSantostefano RL, Forbes H, Eaton S, Hurst JR, Davis K, Smeeth L. Validation of chronic obstructive pulmonary disease recording in the Clinical Practice Research Datalink (CPRD-GOLD). *BMJ open* 2014: 4(7): e005540.
3. Rothnie KJ, Mullerova H, Hurst JR, Smeeth L, Davis K, Thomas SL, Quint JK. Validation of the Recording of Acute Exacerbations of COPD in UK Primary Care Electronic Healthcare Records. *PloS one* 2016: 11(3): e0151357.
4. Oshagbemi OA, Burden AM, Braeken DCW, Henskens Y, Wouters EFM, Driessen JHM, Maitland-van der Zee AH, de Vries F, Franssen FME. Stability of Blood Eosinophils in Patients with Chronic Obstructive Pulmonary Disease and in Control Subjects, and the Impact of Sex, Age, Smoking, and Baseline Counts. *American journal of respiratory and critical care medicine* 2017: 195(10): 1402-1404.
5. Vedel-Krogh S, Nielsen SF, Lange P, Vestbo J, Nordestgaard BG. Blood Eosinophils and Exacerbations in Chronic Obstructive Pulmonary Disease. The Copenhagen General Population Study. *American journal of respiratory and critical care medicine* 2016: 193(9): 965-974.
6. Bafadhel M, Peterson S, De Blas MA, Calverley PM, Rennard SI, Richter K, Fageras M. Predictors of exacerbation risk and response to budesonide in patients with chronic obstructive pulmonary disease: a post-hoc analysis of three randomised trials. *The Lancet Respiratory medicine* 2018: 6(2): 117-126.
7. Quanjer PH, Tammeling GJ, Cotes JE, Pedersen OF, Peslin R, Yernault JC. Lung volumes and forced ventilatory flows. Report Working Party Standardization of Lung Function Tests, European Community for Steel and Coal. Official Statement of the European Respiratory Society. *Eur Respir J Suppl* 1993: 16: 5-40.
8. Bhaskaran K, Smeeth L. What is the difference between missing completely at random and missing at random? *International journal of epidemiology* 2014: 43(4): 1336-1339.
9. Khan NF, Perera R, Harper S, Rose PW. Adaptation and validation of the Charlson Index for Read/OXMIS coded databases. *BMC family practice* 2010: 11: 1.
